# Researches into the Origin of the Morbid State, &c.

**Published:** 1846-01

**Authors:** 


### Art. IX.

*Untersuchungen über Entstehung des Krankheitsgenius, &c. &c.* Von Dr. Martin Geigel, &c. *Würtzburg*, 1840.

*Researches into the Origin of the Morbid State, &c.* By Dr. M. Geigel, Physician at Würtzburg. 8vo, pp. 480.

The systematic collection of facts, the task undertaken by Dr. Geigel, is little less advantageous to science than the discovery of them, especially to medical science, in which the relations of a new fact are almost infinitely numerous. The discovery of some general principle in physiology leads necessarily to a completely new arrangement of the whole cycle of medical studies, just as in war a successful movement is followed by a complete change in the position of the corps d'armée. It is fortunate that labourers are never wanting thus to rearrange details, and group the tried experience of the past around the novelties of the present. The development of the modern solido-humoral pathology is the result of a rearrangement like that here alluded to. The humoral pathology based in a great measure upon experience gave way, in consequence of a natural reaction, to that founded upon general and pathological anatomy. The latter branches of medical science having in their turn been cultivated as far as possible by the simpler means of research, the microscope and a more refined chemical analysis were pressed into the service of the inquirer. A new impulse was thus given to physiology and pathology, and two new lines of research marked out, namely, microscopic anatomy and pathological chemistry, the bearings of which upon medical theory and practice are now beginning to show themselves distinctly, and to point to a fusion of what was valuable in the two theoretical systems mentioned.

The factors of the opposite organic conditions, says Dr. Geigel, are the nervous system and the blood. They have a reciprocal action, and consequently neither an exclusively solid nor exclusively humoral pathology can exhibit the results of such action. If we would estimatethe morbid action of the nervous system on the blood, we must seek out the material changes in the latter ; but we cannot forget on the other hand that the morbid condition of the blood may be the primary cause of these changes. Dr. Geigel's plan includes the consideration of this mutual action and reaction of the blood and nervous system. He first considers the functions of the ganglionic system commencing with an analogy between them, as seen in the fetus and in plants. The influence of the sympathetic on the motion of the blood in the arteries and veins, and on the functions of the absorbents, on digestion, and on the evolution of animal heat are considered. Under a second head the excitants of the ganglionic system are reviewed, and especially the influence which the gases contained in the blood exercise upon them. The following passage will give the reader an idea of Dr. Geigel's views and method.

"Since we know that the blood cannot be vitalized without a nervous system, since we know also that the action of the nervous system cannot be developed without the blood, and, consequently that neither can exhibit life, or develop vital phenomena without the other ; further, since we see that all matters received into the blood act variously, partly on the blood, partly on the nervous system, so matters must absolutely exist in the blood, which shall sometimes exalt, sometimes depress the functions of the nervous system, or produce both results according to the relations which they bear to the blood when received into it. These matters according to my views are the gases contained in the blood. The blood contains, according to the researches of chemists, oxygen, hydrogen, nitrogen, and carbon, the latter generally in combination with oxygen or carbonic acid." (p. 55.)

Dr. Geigel then details experiments showing the relative quantities of these present in arterial and venous blood respectively, and their operations on the nervous system. He infers that carbon paralyses the ganglionic system, but carbonic acid excites the susceptibility of the sensitive nerves. Nitrogen excites the sensitive system, while hydrogen paralyses it, or at least diminishes the irritability of the brain and nerves of sense, acting on the cerebral system as carbon acts on the ganglionic. Oxygen, however, is the primary excitant of the ganglionic system, increasing its activity and irritability. Nitrogen combined with oxygen, as the nitrous oxide, or with hydrogen as ammonia, increases and maintains the activity of the sensitive nerves.

Dr. Geigel then considers the excretory organs as the channels through which these various gases are removed in a solid form from the blood, and then discusses the action of the brain on the ganglionic system, and on the organs of the body generally. In the latter section a variety of physiological details are entered into which we cannot even enumerate.

In the section on the influence of the ganglia on the brain, Dr. Geigel develops a theory of temperaments. The gases in the blood perform a leading part in marking the different temperaments. If oxygen be present in a quantity above par, it determines the sanguineo-choleric temperament; if carbon, we have the melancholic or hypochondriacal. In the next section we have the pathology of fever, and inflammation, discriminating pulmonary inflammation, as it is connected with arterial or venous blood. As the function of the lungs has an important bearing on the changes in the blood, the physiology of sanguification, respiration, and circulation, is minutely considered, especially with reference to the gases and the influence of the nervous system. The succeeding section is devoted to a practical application of the physiological views there developed. Dr. Geigel recommends the use of the electrometer and multiplicator in forming a diagnosis as to the nature of the blood in a patient, and consequently in learning the nature of his disease and the treatment suitable thereto. He thinks these instruments more important than the pleximeter and stethoscope; the data afforded by the latter are only mechanical, those by the former are dynamical. If for example, in a case of rheumatic inflammation of the lungs, the electrometer shows the individual to have negative electricity, or no electricity at all, we know that the crasis of the blood is venous, and as the absorbent power of the veins and lymphatics are diminished under such a condition of the blood, the inflammation must be treated with those remedies which excite the absorbent powers, as nitre, narcotics, mucilaginous medicines. All this, we need not say, is purely hypothetical.

There is a curious section on the laws of absorption from the atmosphere in which the influence on the blood of atmospheric pressure, temperature, purity, and dryness of the atmosphere, the influence of climates and seasons in modifying the composition of the blood and the functions of the nervous and vascular systems, are reviewed. Several pages are then devoted to the pathological applications of the laws thus illustrated, and the theory of epidemics, as cholera and puerperal fever, is discussed. The cholera is, according to our author, a disease dependent on increased venousness, and some space is devoted to a consideration of the epidemic constitution of the years preceding and following the outbreak of that disease. Dr. Geigel also shows the connexion of earthquakes, comets, and other astral and telluric influences on the constitution of the blood, giving it the arterial or venous character as the causes just mentioned, which develop the one or the other, prevailed. The locality and dates of the earthquakes observed in various parts of the world during the twenty-one years subsequent to 1816, are given at length from Professor Schön's researches, (of Wurtzburg.) This connexion of cholera with comets and earthquakes has been noticed by several English writers on the disease; as, for example, by Mr. Orton, whose publication may be considered almost as a standard work.

The remaining third of Dr. Geigel's volume is devoted to a consideration of the pathology and treatment of all the acknowledged epidemics, as ague, hooping-cough, sweating sickness, yellow fever, putrid fever, plague, ganglionic typhus, cholera, &c., scorbutus and rheumatic fever, each finding a place in the list. The hypotheses of our author are applied fully to all these diseases. The cause, for example, of intermittent fever, consists in a predominant supply of hydrogen in the blood. Altogether the book is a curious production, presenting some interesting views, and displaying very considerable research on the part of the author; but displaying also the most dogged and persevering hobby-horsicality we ever witnessed. The blood, blood-gases, and nervous system in all its divisions occupy every page from the first to the last.

